# Systematic review and meta-analysis of initial management of pneumothorax in adults: Intercostal tube drainage versus other invasive methods

**DOI:** 10.1371/journal.pone.0178802

**Published:** 2017-06-22

**Authors:** Min Joung Kim, Incheol Park, Joon Min Park, Kyung Hwan Kim, Junseok Park, Dong Wun Shin

**Affiliations:** 1 Department of Emergency Medicine, Yonsei University College of Medicine, Seoul, Korea; 2 Department of Emergency Medicine, Inje University Ilsan Paik Hospital, Goyang, Korea; National Yang-Ming University, TAIWAN

## Abstract

**Objectives:**

The ideal invasive management as initial approach for pneumothorax (PTX) is still under debate. The purpose of this systematic review and meta-analysis was to examine the evidence for the effectiveness of intercostal tube drainage and other various invasive methods as the initial approach to all subtypes of PTX in adults.

**Methods:**

Three databases were searched from inception to May 29, 2016: MEDLINE, EMBASE, and the Cochrane CENTRAL. Randomised controlled trials that evaluated intercostal tube drainage as the control and various invasive methods as the intervention for the initial approach to PTX in adults were included. The primary outcome was the early success rate of each method, and the risk ratios (RRs) were used for an effect size measure. The secondary outcomes were recurrence rate, hospitalization rate, hospital stay, and complications.

**Results:**

Seven studies met our inclusion criteria. Interventions were aspiration in six studies and catheterization connected to a one-way valve in one study. Meta-analyses were conducted for early success rate, recurrence rate, hospitalization rate, and hospital stay. Aspiration was inferior to intercostal tube drainage in terms of early success rate (RR = 0.82, confidence interval [CI] = 0.72 to 0.95, *I*^*2*^ = 0%). While aspiration and intercostal tube drainage showed no significant difference in the recurrence rate (RR = 0.84, CI = 0.57 to 1.23, I^2^ = 0%), aspiration had shorter hospital stay than intercostal tube drainage (mean difference = -1.73, CI = -2.33 to -1.13, I^2^ = 0%). Aspiration had lower hospitalization rate than intercostal tube drainage, but marked heterogeneity was present (RR = 0.38, CI = 0.19 to 0.76, I^2^ = 85%).

**Conclusion:**

Aspiration was inferior to intercostal tube drainage in terms of early resolution, but it had shorter hospital stay. The recurrence rate of aspiration and intercostal tube drainage did not differ significantly. The efficacy of catheterization connected to a one-way valve was inconclusive because of the small number of relevant studies.

(Registration of study protocol: PROSPERO, CRD42016037866)

## Introduction

Pneumothorax (PTX) is a medical condition marked by the presence of air in the pleural space. It is categorised into two subtypes according to the aetiology: spontaneous PTX and non-spontaneous PTX (i.e. iatrogenic and traumatic). Spontaneous PTX is further classified as primary or secondary according to the presence of underlying lung disease. PTX can also be categorised according to the presence of symptoms: symptomatic PTX and non-symptomatic PTX. Although the exact incidence of PTX cannot be determined, previous studies reported the incidence of primary spontaneous PTX as 7.4–18 per 100,000 males and 1.2–6 per 100,000 females [1,2]. Another study reported the estimated annual emergency admission rates of PTX overall as 16.7 and 5.8 per 100,000 for males and females, respectively [3]. Recurrence after resolution is a major problem in the natural course of PTX, with recurrence rates reported as being between 23 and 37% after the first attack [4,5].

There are two major goals in the treatment of PTX. The immediate goal is to eliminate intrapleural air and re-expand the collapsed lung simultaneously, while the long-term goal is to prevent recurrence. For the achievement of these goals, various non-operative initial treatments have been used in the clinical setting: from the non-invasive method of observation with/without supplemental oxygen, to invasive methods such as intercostal tube drainage (ITD) connected to a suction system, aspiration, and catheterization connected to a one-way valve suction system (Heimlich valve). Of these, the choice of an appropriate therapy is usually based on factors that include the patient’s clinical status, physician’s preference, hospital facilities or policy, and possibility of follow up based on the characteristics of the patient and health system. As a rule, observation without intervention, or only additional supplemental oxygen, are accepted as ideal treatments in cases involving a small PTX accompanied by only minor symptoms or no symptoms. Invasive treatment is usually required as initial management when a PTX is large or accompanied by significant symptoms. Although ITD has been preferred as the standard invasive therapy, recent studies have reported that aspiration and catheterization connected to a one-way valve were also effective for re-expanding a collapsed lung and had similar recurrence rates [6–8].

There have been previous efforts to determine the optimal invasive method. Three systematic reviews compared ITD and aspiration for primary spontaneous PTX in adults [9–11], and one systematic review compared the ambulatory method (i.e. catheter insertion with a one-way valve) with other treatments in adults [12]. To date, there has been no comprehensive review of studies comparing ITD and all other invasive methods for both spontaneous and non-spontaneous PTX. Thus, we performed a systematic review with meta-analysis to seek evidence for the efficiency of ITD and other invasive methods performed at bedside for both spontaneous and non-spontaneous PTX.

## Materials and methods

The study protocol (S1 Protocol) was registered in the international prospective register of systematic reviews (PROSPERO, registration number: CRD42016037866) after permission was granted by the institutional review board of one university hospital. Overall, the process was based on the Cochrane review method [13].

### Literature search

Three databases were searched one reviewer (JMP) from inception to May 29, 2016: MEDLINE, EMBASE, and the Cochrane Central Register of Controlled Trials, with no limits on publication year or language. The search strategy for MEDLINE was developed first and adapted for the other databases (S1 Search strategy). After the initial database search, the reference lists of included studies and relevant reviews were hand-searched as well.

### Selection of studies

Two reviewers (MJK, JMP) independently selected each study for inclusion based on predetermined criteria. Studies were screened by title and abstract first, then by the full text. The inclusion criteria were as follows: (1) studies with adult participants; (2) studies that compared ITD and other invasive methods; (3) studies that included at least one outcome parameter of this work (early success rate, recurrence rate, hospitalization rate, hospital stay, complications) as an outcome; and (4) randomised controlled trials including cluster randomisation. The exclusion criteria were as follows: (1) grey literature sources, such as conference proceedings, theses, and dissertations; and (2) observational studies or controlled clinical trials using a quasi-randomised study design.

### Data extraction

Data from each study were extracted by the two reviewers (MJK, JMP) using a predetermined form. The two reviewers first discussed any disagreement and, if unresolved, consulted with a third reviewer (JP) for a final decision. The following data were extracted from each study: (1) study design; (2) country; (3) number of participants; (4) subtype of PTX; and (5) predetermined outcomes. We attempted to contact the corresponding author of each study by email if any of this information was not provided in the published text.

### Risk of bias assessment

The two reviewers (MJK, JMP) independently assessed the risk of bias using the Cochrane Risk of Bias tool. Reviewers assessed the risk of bias for the six domains with regard to five possible biases and rated each domain as ‘High risk of bias’, ‘Low risk of bias’ or ‘Unclear risk of bias’. Disagreement between the two reviewers was resolved by discussion or consultation with a third reviewer (JP).

### Data analysis

The meta-analysis was performed using Review Manager (RevMan) Version 5.3. (Copenhagen: The Nordic Cochrane Centre, The Cochrane Collaboration, 2014). The primary outcome was early success rate as defined in each study. Recurrence rate (within 1 year), hospitalization rate, hospital stay, and presence of complications were selected as secondary outcomes. Summary statistics for each outcome were obtained by calculating the risk ratio (RR) for dichotomous outcomes and the mean difference for continuous outcomes with a 95% confidence interval (CI) for each. The Mantel–Haenszel method and a random-effects model were used for combining the outcomes of multiple studies, because the treatment protocols for intervention and control varied between studies. For investigating heterogeneity between studies, Higgins’ *I*^2^ statistic was used, with 25%, 50% and 75% considered to indicate low, moderate, and high heterogeneity, respectively [14].

The subgroup analysis was undertaken according to the pre-specified criteria to investigate heterogeneous results or to determine the effect of pre-specified criteria on the pooled estimate. We assumed that clinical difference would mainly originate from the subtypes of PTX, therefore the first episode of primary spontaneous PTX *versus* others (including secondary PTX, more than two episodes, or iatrogenic PTX) was selected as a criterion for the division of subgroups.

## Results

### Selection of studies

**Fig 1:**
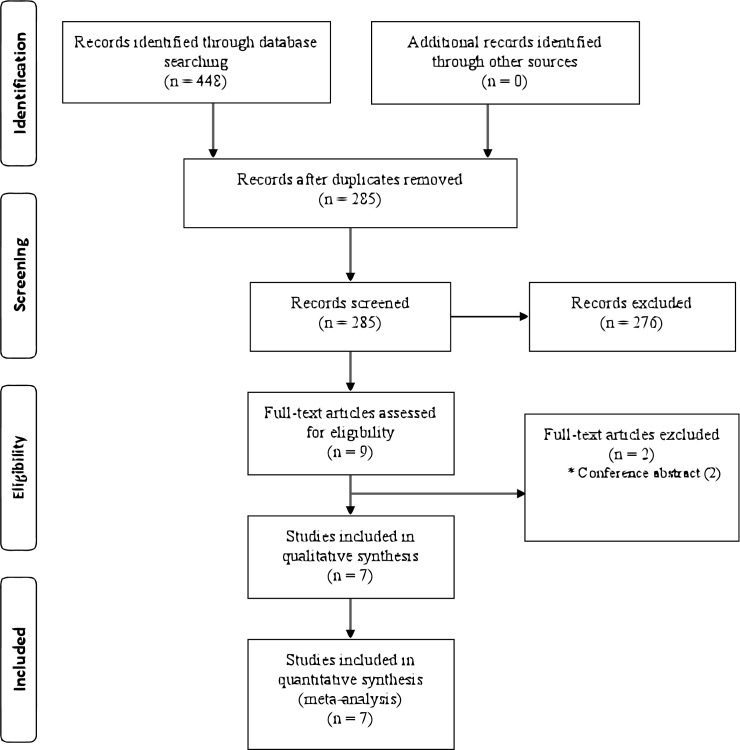
Flow diagram showing selection of studies for review.

In total, 448 studies were identified in the initial comprehensive search; no additional studies were found by manually searching the references of included studies. After excluding duplicate studies, 285 studies were screened by title and abstract, from which 276 inappropriate studies were excluded. Full-text searches were performed for the remaining nine studies, and seven studies were included in the systematic review. Two studies were excluded because they were published as conference proceedings and only abstracts were available [15,16] (Fig 1). We conducted a meta-analysis for the predetermined outcome parameters, except for complications, when they were addressed in more than two studies.

### Study characteristics

Included studies were all individual randomised trials involving 466 participants (224, intervention arm; 242, control arm) (Table 1). Aspiration in six studies and catheterization connected to a one-way valve in one study were adopted as the intervention. Detailed descriptions on the procedures for intervention and control were present, except in two studies where the tube size for ITD was not provided. Five studies enrolled only patients with spontaneous PTX, while two studies enrolled patients with both spontaneous and non-spontaneous PTX. Of the five studies of spontaneous PTX, two studies only included patients with a first episode of primary spontaneous PTX. In the three studies in which PTX other than the first spontaneous episode was included, the proportion of PTX other than the first episode of spontaneous PTX in intervention and control was 22% and 24% in Andrivet et al. [17], 44% and 35% in Parlak et al. [18], 40.9% and 25.9% in Korczynski et al. [19], respectively.

**Table 1:** Characteristics of included studies.

References	Country	Inclusion	Exclusion	I (n)	C (n)	Details of intervention	Details of control	Relevant outcomes
Harvey et al., 1994 [20]	UK	PSP (first episode or recurrent cases)	1. Tension PTX 2. Combined lung disease	35	38	**Aspiration** until no more air was aspirated, the patient was uncomfortable, or three-litre-air was aspirated (16–18 gauge catheter)	**ITD** according to physician’s usual practice (unspecified tube size)	1. Early success rate (only available in intervention group) : no explanation about the definition of early success 2. Recurrence rate (1 year) 3. Hospital stay
Andrivet et al., 1995 [17]	France	PSP, SSP (first episode or first recurrent cases)	1. Posttraumatic or iatrogenic 2. Bilateral PTX 3. Third or more recurrent cases 4. Moderate-to-major associated pleural effusion or haemothorax 5. Contralateral emphysematous bullae 6. Suspected or proven lung cancer, lung abscess, or consolidated pneumonia 7. Diffuse interstitial pneumonitis 8. Body temperature > 38.5°C 9. Moderate-to-severe haemostasis defects 10. Need for mechanical ventilation 11. Prior ipsilateral thoracotomy 12. Suspected or confirmed HIV	33	28	**Aspiration** until cessation of bubbling or for a maximum 30 minutes (16 or 18 Fr catheter; second ICS MCL) : aspiration on Day 3, when poor clinical tolerance was absent* : when immediate aspiration failed, second aspiration was performed 24 hours after initial attempt : if second attempt failed, ITD was applied	**ITD** until 24 hours after no air bubbling followed by clamping the tube for 24 hours and confirmation of lung expansion with x-ray (20 Fr tube; fourth or fifth ICS)	1. Early success rate : complete lung re-expansion (≥80% surface) and no recurrence of a complete PTX within the first 24 hours after last procedure (intervention) : no bubbling within 10 days aspiration period and no short term recurrence requiring secondary insertion (control) 2. Recurrence rate (3 months) 3. Hospital stay
Noppen et al., 2002 [21]	Belgium	PSP (first episode) when symptomatic or greater than 20%	1. Presence of underlying lung disease 2. History of previous PTX 3. Tension PTX	27	33	**Aspiration** until a resistance was felt and air was no longer aspirated (16-gauge catheter; second or third ICS MCL) : when immediate aspiration failed, second aspiration was performed : if second attempt failed, ITD was applied	**ITD** until 24 hours after no air bubbling and confirmation of resolution with X-ray (16 or 18Fr tube; second ICS MCL, fourth or fifth ICS AAL)	1. Early success rate : complete or nearly complete and persistent lung expansion immediately (intervention) : complete lung expansion, absence of air leakage, and chest tube removal within 72 hours after tube placement (control) 2. Recurrence rate (1 year) 3. Hospitalization rate 4. Hospital stay 5. Complications (only available in intervention)
Ayed et al., 2006 [22]	Kuwait	PSP (first episode) when symptomatic or greater than 20%;	1. SSP 2. History of previous PTX 3. Tension PTX 4. Bilateral PTX 5. Iatrogenic PTX 6. Asymptomatic with less than 20% 7. Haemopneumothorax	65	72	**Aspiration** until cessation of bubbling or for a maximum 30 minutes (16-gauge catheter; second ICS MCL) : when immediate aspiration was failed, second aspiration was performed : if second attempt failed, ITD was applied	**ITD** until 24 hours after no air bubbling and confirmation of resolution with X-ray (20 Fr tube; fourth or fifth ICS MAL)	1. Early success rate : complete or nearly complete lung expansion immediately (intervention) : complete lung expansion, absence of air leakage, and chest tube removal within 72 hours after tube placement (control) 2. Recurrence rate (1 year) 3. Hospitalization rate 4. Hospital stay 5. Complications
Parlak et al., 2012 [18]	Netherlands	PSP or traumatic PTX (first episode) when symptomatic or greater than 20%	1. Pregnancy 2. Severe comorbidity 3. Prior randomisation 4. Recurrent or tension PTX 5. Limited decision making 6. Underlying chronic lung disease 7. HIV or Marfan syndrome	25	31	**Aspiration** until a resistance was felt and air was no longer aspirated (1.3-mm catheter in most cases, pneumocatheter in extreme obesity; second or third ICS MCL) :if first attempt failed, ITD was applied	**ITD** until no air leakage with confirmation of lung expansion with X-ray (unspecified tube size; second or third ICS MAL)	1. Early success rate : complete expansion after first attempt with discharge after 24 hours observation (intervention) : expansion of the lung, counteraction of the air leak and removal of the chest tube with discharge within 72 hours (control) 2. Recurrence rate (1 year) 3. Hospitalization rate 4. Hospital stay
Korczynski et al., 2015 [19]	Poland	PSP, SSP (first or recurrent episode)	1. Pregnancy 2. Tension PTX 3. HIV or severe comorbidity 4. Traumatic PTX 5. Recurrent cases within 1 year from the first episode	22	27	**Aspiration** until resistance was felt and air was no longer aspirated. When aspirated air was more than 2000 ml, catheter connected to Heimlich valve (8Fr catheter; second or third ICS MCL) : if unsuccessful 4 hours after initial management, catheter connected to Heimlich valve for further aspiration. : if lung was not re-expanded in 3–5 days, ITD was applied.	**ITD** until no air leakage with confirmation of lung expansion with X-ray (20–24 Fr tube; fourth or fifth ICS between AAL and MAL)	1. Early success rate : complete or nearly complete and persistent lung re-expansion after aspiration and the absence of air leak and removal of catheter within 5 days (intervention) : complete lung re-expansion, absence of air leak, and chest tube removal within 7 days from tube placement (control) 2. Hospitalization rate 3. Hospital stay 4. Complications
Roggla et al., 1996 [23]	Austria	Not specified (PTX with respiratory distress)	1. Major pleural effusion 2. Patients undergoing mechanical ventilation	17	13	**Catheterization connected to Heimlich valve** Placed catheter until 24 hours after expansion confirmed by X-ray (Thoracic vent: 13Fr catheter connected to a one-way valve)	**ITD** until 24 hours after expansion (14 Fr tube)	1. Early success rate : complete or nearly complete re-expansion in 48 hours after drainage (intervention and control) 2. Hospitalization rate 3. Hospital stay 4. Complications

I: intervention; C: control; PSP: primary spontaneous pneumothorax; PTX: pneumothorax; ITD: intercostal tube drainage; SSP: secondary spontaneous pneumothorax; ICS: intercostal space; MCL: mid clavicular line; AXL: anterior axillary line; MAL: mid axillary line

*Criteria for poor clinical tolerance were as follows: systolic blood pressure <90 mmHg or >170 mmHg after 1 hour of bed rest in a previously normotensive patient; diastolic blood pressure >110 mmHg; heart rate >130/min on arrival or 110/min after 1 hour of bed rest; respiratory rate >35/min on arrival or >25/min after 1 hour of bed rest; arterial oxygen saturation <85% with room air or <90% with supplemental oxygen of 3L/min via nasal prongs; arterial pH <7.35; diaphoresis, agitation, or encephalopathy.

The study by Andrivet et al. [17] consisted of two different study designs: a randomised controlled trial comparing aspiration and ITD, and an open non-randomised trial performing aspiration on all included patients to observe the effect of aspiration. We analysed only the randomised controlled trial data from this study. Another unique characteristic of this study was that the intervention arm was divided into two groups: immediate aspiration and delayed aspiration. Aspiration was performed immediately in patients with poor clinical tolerance and on Day 3 when poor clinical tolerance was absent. In the study by Korczynski et al. [19], when initial aspiration did not achieve successful lung expansion, a Heimlich valve was applied for additional aspiration.

### Risk of bias assessment

Appropriateness of the method for random sequence generation was adapted from each study except for two (Harvey et al. [20] and Andrivet et al. [17]) in which these details were not addressed (Table 2). Allocation concealment was secured by a separate random number list using a computer-generated numerical table in the study by Noppen et al. [21], and it was not secured in the study by Ayed et al. [22] because envelopes used in the study were not reported as opaque or sequentially numbered; the other studies did not address this detail and were rated as ‘Unclear’. Consequently, the risk for selection bias was low only in the study by Noppen et al. [21]. The risk domain regarding blinding was rated as ‘High’ for all of the studies because blinding could not be secured in all of the studies. The risk of bias for incomplete outcome data was low in all included studies because all of the participants were followed to the main outcome (i.e. early success rate). The risk of bias for selective reporting was secured only in the study by Parlak et al. [18], which followed a pre-registered study protocol.

**Table 2:** Risk of bias assessment.

References	Random sequence generation	Allocation concealment	Blinding of participants and personnel	Blinding of outcome assessment	Incomplete outcome data	Selective reporting
Harvey et al. [20]	Unclear	Unclear	High	High	Low	Unclear
Andrivet et al. [17]	Unclear	Unclear	High	High	Low	Unclear
Noppen et al. [21]	Low	Low	High	High	Low	Unclear
Ayed et al. [22]	Low	High	High	High	Low	Unclear
Parlak et al. [18]	Low	Unclear	High	High	Low	Low
Korczynski et al. [19]	Low	Unclear	High	High	Low	Unclear
Roggla et al. [23]	Low	Unclear	High	High	Low	Unclear

### Primary outcome

**Fig 2:**
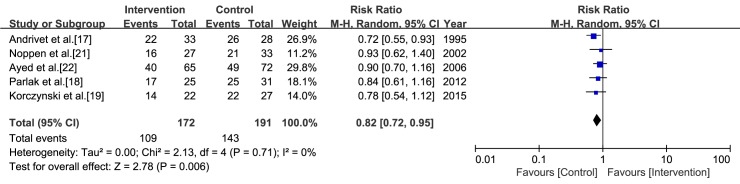
Meta-analysis of the early success rate associated with the aspiration versus intercostal tube drainage. The risk ratio for early success rate was used for effect size measure. The Mantel-Haenzel method and a random-effects model were used for calculating the pooled estimate.

In the study by Harvey et al. [20], the early success rate for the control arm was not reported. Therefore, we calculated a pooled estimate from the five studies in which aspiration was performed as the intervention (Fig 2). The early success rates for aspiration and ITD were 63.4% (109/172) and 74.9% (143/191), respectively. From the meta-analysis, aspiration was inferior to ITD in terms of early success rate (RR = 0.82, CI = 0.72 to 0.95, *I*^*2*^ = 0%). The early success rate did not differ significantly between catheterization connected to a one-way valve and ITD (RR = 1.04, CI = 0.78 to 1.39) [22].

#### Subgroup analysis with the PTX subtype in inclusion of each study

**Fig 3:**
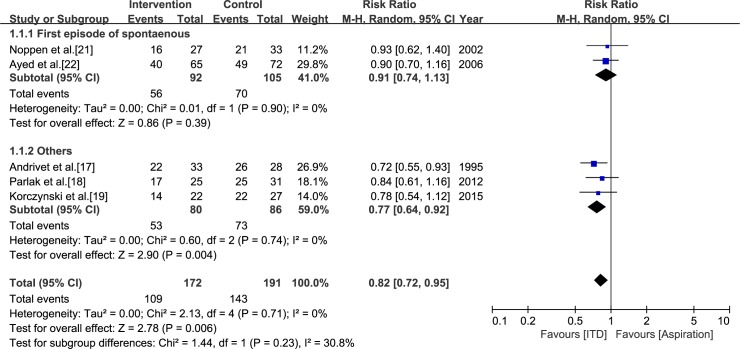
Subgroup analysis of the studies that compared aspiration and intercostal tube drainage. The subgroupings were assigned according to the subtype of pneumothorax included in each study; i.e., first episode of primary spontaneous pneumothorax versus other types.

There was no significant difference between aspiration and ITD in the pooled estimate of two studies in which all participants had a first episode of primary spontaneous PTX (RR = 0.91, CI = -0.74 to 1.13, *I*^*2*^ = 0%) [21,22]. On the other hand, the pooled estimate of three studies in which patients with PTX other than a first spontaneous episode were included showed a benefit of ITD for early success (RR = 0.77, CI = -0.64 to 0.92, *I*^*2*^ = 0%) [17–19]. The difference between the two subgroups showed moderate heterogeneity (*I*^*2*^ = 30.8%) (Fig 3).

### Secondary outcomes

#### Recurrence rate

**Fig 4:**
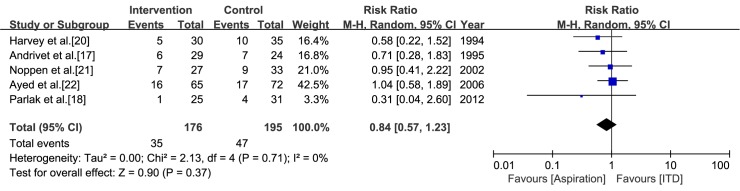
Meta-analysis of the recurrence rate within 1 year associated with aspiration versus intercostal tube drainage.

Most studies reported recurrent cases within 1 year [18, 20–22], but recurrent cases within 3 months were reported in the study by Andrivet et al. [17]. A pooled estimate of the recurrence rate indicated that no significance difference exists between aspiration and ITD (RR = 0.84, CI = 0.57 to 1.23, *I*^*2*^ = 0%) (Fig 4).

#### Hospitalization rate

**Fig 5:**
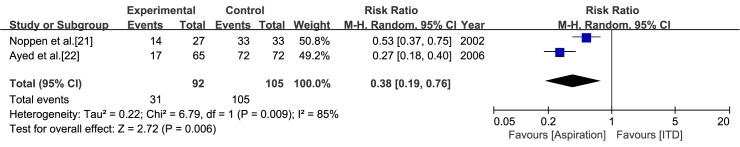
Meta-analysis of the hospitalization rate associated with aspiration versus intercostal tube drainage.

Two of the studies using aspiration as the intervention did not address the hospitalization rates for both the intervention and control arms [17, 20]. Of the other four studies using aspiration as the intervention, we excluded the studies by Parlak et al. [18] and Korczynski et al. [19] because all of the participants in both the intervention and control arms were hospitalized for X-ray confirmation on Day 2. A pooled estimate of the remaining two studies [21, 22] in which aspiration was the intervention showed lower risk for hospitalization with marked heterogeneity (RR = 0.38, CI = 0.19 to 0.76, *I*^*2*^ = 85%) (Fig 5). In the study by Roggla et al. [23], catheterization connected to a one-way valve enabled ambulation such that a significant portion of patients (12 of 17) allocated to intervention was treated on outpatient basis.

#### Hospital stay

**Fig 6:**
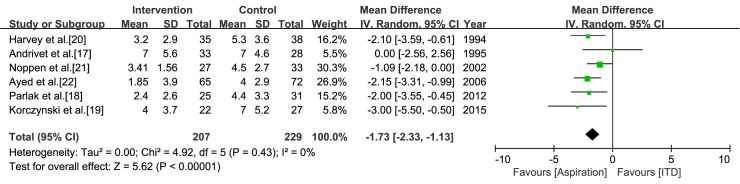
Meta-analysis of the hospital stay associated with aspiration versus intercostal tube drainage.

In the study by Korczynski et al. [19], the hospital stays for both the intervention and control arms were given as median value and interquartile range. Thus, we imputed the standard deviation by dividing the interquartile range by 1.35 [13]. In the meta-analysis of six studies in which aspiration was the intervention, the pooled estimate showed a shorter hospital stay for aspiration (mean difference = -1.73, CI = -2.33 to -1.13, *I*^*2*^ = 0%) (Fig 6). In the study by Roggla et al. [22], the hospital stay (mean±standard deviation) for the intervention and control arms was 4± 3.5 days and 8± 6.2 days, respectively (not significant).

#### Complications

Overall, reported complications in both the intervention and control arms of each study were rare (Table 3). Among studies that compared aspiration and ITD, only two collected data about complications in both intervention and control arm [19,22]. No complications were reported in any of the two arms in the study by Korczynski et al. [19], but there was one complication from aspiration and three complications from ITD in the study by Ayed et al. [22]. In the study by Roggla et al. [23], there were seven complications from catheterization connected to a one-way valve and three from ITD.

**Table 3:** Complications in the intervention and control arms of each study.

References	Aspiration	ITD
Harvey et al. [20]	NR	NR
Andrivet et al. [17]	NR	NR
Noppen et al. [21]	0	NR
Ayed et al. [22]	Subcutaneous emphysema (1)	Subcutaneous emphysema (2) Tube blockage (2) Exit site infection (1)
Parlak et al. [18]	NR	NR
Korczynski et al. [19]	0	0
	**Catheterization connected to a one-way valve**	**ITD**
Roggla et al. [23]	Skin emphysema (3) Primary malposition (1) Secondary dislocation (3)	Skin emphysema (3)

ITD: intercostal tube drainage; NR: not reported.

## Discussion

Although various approaches for the management of PTX have been proposed, there is no consensus on the optimal initial choice. Attempts to generate an international consensus on the optimal management of spontaneous PTX include suggested guidelines published by the American College of Chest Physicians in 2001, the Belgian Society of Pulmonology in 2005, and the British Thoracic Society in 2010 [24–26]. All three guidelines adopted clinical factors (e.g. patient’s symptoms, PTX size, or PTX subtype) as the main determinants for optimal selection of management approach and agreed upon a conservative approach (observation or supplemental oxygen) for asymptomatic, small-size PTX. However, the details of these guidelines diverge in cases where an invasive intervention is warranted for re-expansion of a collapsed lung. The American College of Chest Physicians recommend ITD primarily, but the British Thoracic Society gives priority to aspiration for both primary and secondary spontaneous PTX. The Belgian Society of Pulmonology guidelines recommend either aspiration or small-bore catheterization with a Heimlich valve attachment or underwater seal for evacuating intrapleural air in primary spontaneous PTX, and ITD in secondary spontaneous PTX.

For this review, we primarily aimed to identify studies that compared ITD and all other invasive methods available at bedside for the management of PTX irrespective of subtype, i.e. primary and secondary spontaneous PTX, and non-spontaneous PTX. In a recent systematic review, the literature search was confined to the three existing studies that included cases of spontaneous PTX only and for which the intervention was aspiration [11]. By contrast, our literature search revealed that two invasive methods have been compared with ITD for the management of PTX. Ultimately, this review included six randomised controlled trials in which the intervention was aspiration, and one trial in which the intervention was catheterization connected to a one-way valve. Meta-analyses were available only for the six studies that compared aspiration and ITD.

Overall, the pooled estimate of the RR for early success indicated that ITD is more effective than aspiration, which coincides with the results of a previous review by Aguinagalde et al. [11]. Although the statistical heterogeneity between studies proved to be very low, differences in detailed characteristics between individual studies might have influenced the pooled estimate. In subgroup analysis, moderate heterogeneity between subgroups indicates that the effectiveness of the two interventions might be different according to PTX subtype. However, considering that the subgroup analysis is observational by nature, and the number of studies within each subgroup is small, further prospective controlled trials might be required to ascertain which method is more appropriate for early success in each PTX subtype.

Unlike the other studies, in which aspirations were performed immediately in all patients, in the study by Andrivet et al. [17] aspiration was delayed to Day 3 in 26 of 33 patients allocated to the intervention arm (78.8%). In that study, delayed aspiration might have had a negative influence on the early success rate, possibly due to PTX progression during the observation period before aspiration. To evaluate the effect of this study on the pooled estimate, we performed a post-hoc sensitivity analysis. When the results of this study were removed, ITD was still favoured although a statistical significance was not guaranteed (RR = 0.87, CI = 0.74 to 1.02, *I*^*2*^ = 0%).

Theoretically, an inserted chest tube can irritate the pleura, promoting pleural symphysis; thus, ITD might have less risk for recurrence than aspiration [17]. However, our work revealed that there was no significant difference between aspiration and ITD in the recurrence rate within 1 year. In the study by Andrivet et al. [17], recurrence was followed for only 3 months after the event unlike the 1-year follow-up period in the other included studies. To evaluate the effect of this study on the pooled estimate, we conducted a post-hoc sensitivity analysis by summing the RR for recurrence excluding this study. The resulting pooled estimate still did not show a significant difference between aspiration and ITD (RR = 0.87, CI = 0.57 to 1.32, *I*^*2*^ = 0%). This reinforces the conclusion drawn from the pooled estimate of all five studies.

For combining the RRs for hospitalization, two studies showed a significantly lower hospitalization rate when aspiration was applied [21, 22]. However, the number of included studies was insufficient, and marked heterogeneity was present; thus, determination of a favoured method on this outcome was inconclusive.

Length of stay was significantly shorter in participants who received aspiration compared to ITD. Combination of this outcome did not show heterogeneity. As aforementioned, in the study by Andrivet et al. [17], the delay in aspiration for a significant portion of the intervention arm (78.8%) might have lengthened their stay. When the results of that study are excluded, the pooled estimate showed a greater difference without heterogeneity (mean difference = -1.83, CI = -2.45 to -1.21, *I*^*2*^ = 0%).

The risk of selection bias in the methodological quality of the included studies was problematic. Although each study claimed that all of their participants were randomly allocated to each treatment arm, details about randomisation and allocation concealment were not readily available. In spite of this deficiency, we conducted the meta-analysis because each study addressed comparability between their intervention and control. Although blinding was not secured in all studies considering the characteristics of the offered treatment, we believe that it probably did not lead to performance bias or detection bias. Among the three previous systematic reviews on the same topic, the study by Wakai et al.[10] assessed only the risk of bias regarding randomisation and blinding, and other studies used other tools for assessment [9,11]. Thus, comparing their results with ours was not fully applicable. However, their assessments were in agreement with ours for the same risk of bias.

Our work had some limitations. First, although we executed a subgroup analysis according to pre-specified criteria (i.e. PTX subtype), other factors that might pose clinical heterogeneity were not evaluated, including differences in patient severity and practitioner competence, and details in the application of procedures; however, such information was not accessible. Moreover, considering the low number of included studies, multiple subgroup analyses according to various factors might have led to false-positive results. Second, the results of two randomised controlled trials that were published as conference proceedings in abstract form only were not combined because they corresponded to pre-specified exclusion criteria, and an assessment of their quality was impossible. Third, all included studies had a relatively small number of participants. Lastly, because of the small number of included studies, the possibility of publication bias could not be assessed. However, we conducted an additional search for appropriate ongoing clinical trials in ‘ClinicalTrials.gov’, and none were found.

## Conclusion

In this review, we evaluated studies comparing the effectiveness of ITD to two invasive methods (aspiration and catheterization connected to a one-way valve) for the initial approach in the management of PTX. While ITD was more effective than aspiration in the early resolution of PTX, the recurrence rate within 1 year did not differ between the initial approaches, and the length of stay was shorter with aspiration. Overall, at present, there is insufficient evidence to determine the efficacy and safety of aspiration versus ITD in the management of PTX in adults. The efficacy of catheterization connected to a one-way valve was inconclusive because of an insufficient number of studies. In the future, primary studies in which factors of clinical heterogeneity are well controlled, followed by a meta-analysis of a large number of such studies, will be warranted for a conclusive determination of the optimal initial invasive approach to PTX.

## Differences between protocol and review

In the initial protocol, studies involving neonates were excluded. However, PTX in paediatric patients might be quite different from that in adults in terms of pathophysiology, treatment, and prognosis. Therefore, a separate analysis of PTX in children may be more appropriate. Since we anticipated that we would find very few studies that included only paediatric patients or investigated them separately, in the early stage of our study, we decided to include in our analysis studies that investigated adult patients only. In the revised protocol, inclusion of paediatric patients was designated as an exclusion criterion.

A subgroup analysis according to intervention methods was originally planned. However, because only two intervention methods were evaluated in previous studies and the characteristics of each intervention were too different to sum up their effect sizes, we decided not to sum the effect sizes of the two methods. Instead, we decided to report the effect size of each method separately. The protocol was revised accordingly.

Although at the time of registration of the protocol, we thought that we would be able to acquire funding, eventually, we could not. Thus, we changed the funding-related information in the revised protocol.

## Supporting information

S1 Checklist PRISMA checklist. (DOC)

S1 Search strategy Search strategy of three databases. (DOCX)

S1 Protocol Study protocol. (PDF)
