# Supplementary material for: Systematic review and meta-analysis of initial management of pneumothorax in adults: Intercostal tube drainage versus other invasive methods
Source: PLoS One. 2017 Jun 22;12(6):e0178802. doi: 10.1371/journal.pone.0178802 (PMC5480863; doi:10.1371/journal.pone.0178802)
Supplement: S1 Search strategy — (DOCX) [file pone.0178802.s002.docx]

A. MEDLINE

1. "Pneumothorax"[Mesh]

2. "pneumothorax"[TW] OR "Tension Pneumothorax"[TW] OR "Pneumothorax, Tension"[TW] OR "Pneumothoraxs, Tension"[TW] OR "Tension Pneumothoraxs"[TW] OR "Pressure Pneumothorax"[TW] OR "Pneumothorax, Pressure"[TW] OR "Pneumothoraxs, Pressure"[TW] OR "Pressure Pneumothoraxs"[TW] OR "Primary Spontaneous Pneumothorax"[TW] OR "Pneumothoraxs, Primary Spontaneous"[TW] OR "Primary Spontaneous Pneumothoraxs"[TW] OR "Spontaneous Pneumothorax, Primary"[TW] OR "Spontaneous Pneumothoraxs, Primary"[TW] OR "Spontaneous Pneumothorax"[TW] OR "Pneumothorax, Spontaneous"[TW] OR "Pneumothoraxs, Spontaneous"[TW] OR "Spontaneous Pneumothoraxs"[TW]

3. 1 OR 2

4. "Chest Tubes"[Mesh]

5. "Chest Tube"[TW] OR "Tube, Chest"[TW] OR "Tubes, Chest"[TW] OR "Chest Tubes"[TW]

6. "Thoracostomy"[Mesh]

7. Thoracostomies[TW] OR "Needle Thoracostomy"[TW] OR "Needle Thoracostomies"[TW] OR "Thoracostomies, Needle"[TW] OR "Thoracostomy, Needle"[TW] OR thoracostomy[TW]

8. 4 OR 5 OR 6 OR 7

9. 3 AND 8

10. (randomized controlled trial [pt] OR controlled clinical trial [pt] OR randomized controlled trials [mh] OR random allocation [mh] OR double-blind method [mh] OR single-blind method [mh] OR clinical trial [pt] OR clinical trials [mh] OR "clinical trial" [tw] OR ((singl* [tw] OR doubl* [tw] OR trebl* [tw] OR tripl* [tw]) AND (mask* [tw] OR blind* [tw])) OR placebos [mh] OR placebo* [tw] OR random* [tw] OR research design [mh:noexp]) NOT (animals [mh] NOT human [mh])

11. 9 AND 10

12. 9 /Filters: Randomized Controlled Trial

13. 11 OR 12

B. EMBASE

1. 'pneumothorax'/exp OR pneumothorax

2. 'bilateral pneumothorax'/exp OR 'bilateral pneumothorax' OR 'chronic pneumothorax'/exp OR 'chronic pneumothorax' OR 'pneumothorax, chronic'/exp OR 'pneumothorax, chronic' OR 'pneumothorax, valvular'/exp OR 'pneumothorax, valvular' OR 'valvular pneumothorax'/exp OR 'valvular pneumothorax' OR ‘pneumothorax’ OR ‘Tension Pneumothorax’ OR ‘Pneumothorax, Tension’ OR ‘Pneumothoraxs, Tension’ OR ‘Tension Pneumothoraxs’ OR ‘Pressure Pneumothorax’ OR ‘Pneumothorax, Pressure’ OR ‘Pneumothoraxs, Pressure’ OR ‘Pressure Pneumothoraxs’ OR ‘Primary Spontaneous Pneumothorax’ OR ‘Pneumothoraxs, Primary Spontaneous’ OR ‘Primary Spontaneous Pneumothoraxs’ OR ‘Spontaneous Pneumothorax, Primary’ OR ‘Spontaneous Pneumothoraxs, Primary’ OR ‘Spontaneous Pneumothorax’ OR ‘Pneumothorax, Spontaneous’ OR ‘Pneumothoraxs, Spontaneous’ OR ‘Spontaneous Pneumothoraxs’

3. 1 OR 2

4. 'chest tube'/exp OR 'chest tube'

5. ‘chest drain’/exp OR ‘chest drain’ OR ‘chest drain (physical object)’/exp OR ‘chest drain (physical object)’ OR ‘chest tubes’/exp OR ‘chest tubes’ OR ‘intercostal drain’/exp OR ‘intercostal drain’ OR ‘PleuraFlow’/exp OR ‘PleuraFlow’ OR ‘tube, chest’/exp OR ‘tube, chest’ OR ‘tubes, chest’/exp OR ‘tubes, chest’

6. 'thorax drainage'/exp OR 'thorax drainage'

7. ‘chest drainage’/exp OR ‘chest drainage’ OR ‘stomy, thoraco’/exp OR ‘stomy, thoraco’ OR ‘thoracic drainage’ OR ‘thoracic drainage’ OR ‘thoracostomy’/exp OR thoracostomy OR Thoracostomies OR ‘Needle Thoracostomy’ OR ‘Needle Thoracostomies’ OR ‘Thoracostomies, Needle’ OR ‘Thoracostomy, Needle’

8. 4 OR 5 OR 6 OR 7

9. 3 AND 8

10. 9 AND [randomized controlled trial]/lim AND [embase]/lim

C. Cochran Central Register of Controlled Trials

1. [mh pneumothorax]

2. "pneumothorax":ti,ab,kw OR "Tension Pneumothorax":ti,ab,kw OR "Pneumothorax, Tension":ti,ab,kw OR "Pneumothoraxs, Tension":ti,ab,kw OR "Tension Pneumothoraxs":ti,ab,kw OR "Pressure Pneumothorax":ti,ab,kw OR "Pneumothorax, Pressure":ti,ab,kw OR "Pneumothoraxs, Pressure":ti,ab,kw OR "Pressure Pneumothoraxs":ti,ab,kw OR "Primary Spontaneous Pneumothorax":ti,ab,kw OR "Pneumothoraxs, Primary Spontaneous":ti,ab,kw OR "Primary Spontaneous Pneumothoraxs":ti,ab,kw OR "Spontaneous Pneumothorax, Primary":ti,ab,kw OR "Spontaneous Pneumothoraxs, Primary":ti,ab,kw OR "Spontaneous Pneumothorax":ti,ab,kw OR "Pneumothorax, Spontaneous":ti,ab,kw OR "Pneumothoraxs, Spontaneous":ti,ab,kw OR "Spontaneous Pneumothoraxs":ti,ab,kw

3. 1 OR 2

4. [mh "Chest Tubes"]

5. "Chest Tube":ti,ab,kw OR "Tube, Chest":ti,ab,kw OR "Tubes, Chest":ti,ab,kw OR "Chest Tubes":ti,ab,kw

6. [mh Thoracostomy]

7. "Thoracostomies":ti,ab,kw OR "Needle Thoracostomy":ti,ab,kw OR "Needle Thoracostomies":ti,ab,kw OR "Thoracostomies, Needle":ti,ab,kw OR "Thoracostomy, Needle":ti,ab,kw OR thoracostomy:ti,ab,kw

8. 4 OR 5 OR 6 OR 7

9. 3 AND 8
