# Supplementary material for: Systematic review and meta-analysis of initial management of pneumothorax in adults: Intercostal tube drainage versus other invasive methods
Source: PLoS One. 2017 Jun 22;12(6):e0178802. doi: 10.1371/journal.pone.0178802 (PMC5480863; doi:10.1371/journal.pone.0178802)
Supplement: S1 Protocol — (PDF) [file pone.0178802.s003.pdf]

Conventional tube thoracostomy and other invasive interventions for pneumothorax in adults: systematic review and meta-analysis of randomized controlled trials

Min Joung Kim, Incheol Park, Junseok Park, Kyung Hwan Kim, Dong wun Shin

Citation
Min Joung Kim, Incheol Park, Junseok Park, Kyung Hwan Kim, Dong wun Shin. Conventional tube thoracostomy and other invasive interventions for pneumothorax in adults: systematic review and meta-analysis of randomized controlled trials. PROSPERO 2016:CRD42016037866 Available from http://www.crd.york.ac.uk/PROSPERO_REBRANDING/display_record.asp?ID=CRD42016037866

Review question(s)
Which method shows higher early success rate for the treatment of pneumothorax? conventional tube thoracostomy vs. other invasive methods.

Which method has shorter hospital stay for the patients with pneumothorax? conventional tube thoracostomy vs. other invasive method.

Which method has lower hospital admission rate for the patients with pneumothorax? conventional tube thoracostomy vs. other invasive method.

Which method has less complication for the treatment of pneumothorax? conventional tube thoracostomy vs. other invasive method.

Which method has lower recurrence rate for the treatment of pneumothorax? conventional tube thoracostomy vs. other invasive method.

Searches
We search three core databases (MEDLINE, EMBASE, Cochrane library) first, and then the references of included studies and relevant reviews are hand-searched.

There is no restriction on language or publication period.

Types of study to be included
Inclusion criteria: randomized controlled trials including cluster-randomized trials

Exclusion criteria:

observational studies, controlled clinical trials such as quasi-randomized trials

Condition or domain being studied
Spontaneous and traumatic pneumothorax

Participants/ population
Inclusion criteria: adult patients with spontaneous and traumatic pneumothorax

Exclusion criteria: pediatric patients including new born

Intervention(s), exposure(s)
Inclusion criteria: Use of invasive techniques other than conventional tube thoracostomy (for example, Needle aspiration, aspiration via small bore catheter, thoracic vent)

Exclusion criteria: surgical method (thoracotomy, video assisted thoracoscopic surgery)

Comparator(s)/ control
Inclusion criteria: use of conventional tube thoracostomy connected to a drainage system

Outcome(s)
Primary outcomes
early success rate

The details of early success complies with the definition used in each study.

Secondary outcomes
1. Hospital admission rate

2. Length of stay for admission

3. Complication rate

4. Recurrence rate (within one year)

The details of each secondary outcome complies with the definition used in each study.

Risk of bias (quality) assessment
The two reviewers independently validated the quality of each included study using the Cochrane Risk of Bias tool. Reviewers assessed the risk of bias for the six domains regarding five possible biases and rated each domain as ‘High risk of bias’, ‘Low risk of bias’, or ‘Unclear risk of bias’. Disagreement between two reviewers was resolved by discussion or consultation with a third reviewer.

Strategy for data synthesis
The Mantel–Haenszel method and a random-effects model were used for combining results of included studies. To avoid unit-of-analysis error in cluster randomized controlled trials, numbers of whole and failed participants at first attempt in each study were adjusted by dividing with the design effect.

Analysis of subgroups or subsets
According to the subtype of pneumothorax: first spontaneous vs. others

Contact details for further information
Joon Min Park

411-706 Emergency department, Inje University Ilsanpaik hospital, Juhwa-ro 170, Ilsanseo-gu, Goyang-si, Gyeonnggi-do, Korea

aero7@outlook.kr

Organisational affiliation of the review
none

Review team
Professor Min Joung Kim, Department of Emergency Medicine, Yonsei University College of Medicine
Professor Incheol Park, Department of Emergency Medicine, Yonsei University College of Medicine
Professor Junseok Park, Department of Emergency Medicine, Inje University Ilsanpaik hospital
Professor Kyung Hwan Kim, Department of Emergency Medicine, Inje University Ilsanpaik hospital
Professor Dong wun Shin, Department of Emergency Medicine, Inje University Ilsanpaik hospital

Anticipated or actual start date
29 May 2016

Anticipated completion date
31 March 2017

Funding sources/sponsors
none

Conflicts of interest
None known

Language
English

Country
South Korea

Subject index terms status
Subject indexing assigned by CRD

Subject index terms
Chest Tubes; Humans; Paracentesis; Pneumothorax; Randomized Controlled Trials as Topic; Thoracostomy; Thoracotomy

Stage of review
Ongoing

Date of registration in PROSPERO
31 May 2016

Date of publication of this revision
27 March 2017

Stage of review at time of this submission	Started	Completed
Preliminary searches	Yes	Yes
Piloting of the study selection process	Yes	Yes
Formal screening of search results against eligibility criteria	Yes	Yes
Data extraction	Yes	Yes
Risk of bias (quality) assessment	Yes	Yes
Data analysis	Yes	Yes

PROSPERO
International prospective register of systematic reviews
The information in this record has been provided by the named contact for this review. CRD has accepted this information in good faith and registered the review in PROSPERO. CRD bears no responsibility or liability for the content of this registration record, any associated files or external websites.
